# A Review on the Therapeutic Role of TKIs in Case of CML in Combination With Epigenetic Drugs

**DOI:** 10.3389/fgene.2021.742802

**Published:** 2021-10-22

**Authors:** Mohd Amir, Saleem Javed

**Affiliations:** Department of Biochemistry, Faculty of Life Sciences, Aligarh Muslim University, Aligarh, India

**Keywords:** chronic myeloid leukemia, tyrosine kinase inhibitors, imatinib, epigenetic, DNMT inhibitors, HDAC inhibitors, TKI resistance

## Abstract

Chronic myeloid leukemia is a malignancy of bone marrow that affects white blood cells. There is strong evidence that disease progression, treatment responses, and overall clinical outcomes of CML patients are influenced by the accumulation of other genetic and epigenetic abnormalities, rather than only the BCR/ABL1 oncoprotein. Both genetic and epigenetic factors influence the efficacy of CML treatment strategies. Targeted medicines known as tyrosine-kinase inhibitors have dramatically improved long-term survival rates in CML patients during the previous 2 decades. When compared to earlier chemotherapy treatments, these drugs have revolutionized CML treatment and allowed most people to live longer lives. Although epigenetic inhibitors’ activity is disrupted in many cancers, including CML, but when combined with TKI, they may offer potential therapeutic strategies for the treatment of CML cells. The epigenetics of tyrosine kinase inhibitors and resistance to them is being studied, with a particular focus on imatinib, which is used to treat CML. In addition, the use of epigenetic drugs in conjunction with TKIs has been discussed. Resistance to TKIs is still a problem in curing the disease, necessitating the development of new therapies. This study focused on epigenetic pathways involved in CML pathogenesis and tumor cell resistance to TKIs, both of which contribute to leukemic clone breakout and proliferation.

## Chronic Myeloid Leukemia

The malignancy of white blood cells in chronic myeloid leukemia (CML), often referred to as chronic myelogenous leukemia. CML is a clonal myeloproliferative disease that affects over 15% of adults and the average age for the outbreak of this disease is 57 years (Hochhaus et al., 2020; Jabbour and Kantarjian, 2020). CML is more prevalent in men than in women (ratio 1.4:1), accounting for 15–25% of all juvenile leukemias and 14% of all leukemias in the countries lying towards the west. The Philadelphia chromosome (Ph), which occurs between chromosomes 9 and 22, is the characteristic feature of CML and has been regarded to be the first malignancy related to genetic abnormalities. This translocation results in the formation of the BCR-ABL1 hybrid gene, which expresses a constitutively active oncokinase protein. The ABL kinase domain that spans around 300 amino acids, has a bilobed conformation with a C-terminal and an N-terminal lobe, with ATP binding in a cleft flanked by these lobes. Because of the flexibility of C-terminal loop, which contains a “DFG”-motif (Asp381-Phe382-Gly383), the kinase can be in an active (open) or inactive (closed) conformation (Reddy and Aggarwal, 2012; Balabanov et al., 2014). CML is classified into three phases namely chronic phase (CP), accelerated phase (AP) and blast crisis (BC). The presence of one of the various hematological (like persistent thrombocytopenia) or provisional (like hematological resistance to the drug) criteria for responding to tyrosine kinase inhibitor (TKI) defines AP phase, as per WHO criteria (Arber et al., 2016).

## Targeted Therapy for CML Using Tyrosine Kinase Inhibitors

### Current Use of TKIs for CML Treatment

TKIs are small molecules that are used to treat a wide range of malignancies, including leukemia and solid tumors (Amir et al., 2020). TKIs have been developed as a targeted treatment for BCR-ABL1 kinase activity suppression (Cilloni and Saglio, 2012). TKI therapy for CML patients has resulted in a substantial improvement in disease outcomes and a near-normal lifespan. CML is a well-studied TK-dependent alteration model disease. As a consequence of fundamental and clinical research in clinical practice, TKIs are currently the benchmark in the treatment of CML patients and an effective example of targeted therapy (Balabanov et al., 2014). Imatinib, for example, is one of the first TKIs to be certified for the treatment of CML and is presently used as a first-line treatment, while nilotinib and dasatinib are the next discoveries (Özgür Yurttaş and Eşkazan, 2020).

The first generation TKI binds to an inactive form of BCR-ABL conformation, while second generation TKIs show binding affinity for both the active and inactive forms. The TKIs of second generation (dasatinib, nilotinib, bosutinib) are approximately 1,000 times more sensitive than imatinib in terms of efficacy against BCR-ABL resistant mutations (Kantarjian et al., 2010; Saglio et al., 2010). The binding of imatinib with BCR-ABL kinase in its ATP-binding pocket is hampered by T315I mutation, which is the result of a substitution of isoleucine for threonine at position 315 of the amino acid chain. Approximately 12 percent of adult patients with BCR-ABL leukemia have seen this mutation, and is more common in people with severe conditions. However, dasatinib and nilotinib against T315I are not effective (Soverini et al., 2006; Soverini et al., 2007).

### The Survival Rate and Long Term Response in CML Patients in the Era of TKI Therapy

The survival has improved significantly to the point the survival in patients with CML is approaching that of the normal population with access to TKI therapy. Patients with newly diagnosed CML-CP had an OS rate that is only marginally lower than the normal population when treated with TKIs. Patients who obtained CCyR or better, on the other hand, have an OS rate that is comparable to the general population. This highlights the need of ensuring the best possible care for CML patients of all ages to maximize the benefits of these life-changing treatments (Sasaki et al., 2015). The long-term response of patients treated with different TKI therapies and their outcomes were examined by Jain et al. using Kaplan-Meier method. Patients treated with imatinib 800, dasatinib, or nilotinib had better long-term responses and outcomes than those treated with imatinib 400. Transformation to AP and BC phases, as well as mortality from CML, are now uncommon with current TKI therapy methods when well managed. However, discontinuation of treatment was more prevalent in imatinib patients than in TKIs of the second generation (Jain et al., 2015).

In the era of TKIs, CML-CP patients who lived for a certain number of years had good clinical results. Patients may, however, quit medication for some reason throughout time, affecting OS. It’s worth noting that the good conditional long-term outcomes predicted are based on rigorous ongoing monitoring of patients for CML and other comorbidities, as well as side events related to TKI usage, as done in clinical studies. It is not reasonable to believe that this may be generalized to situations where patients are treated less strictly (Sasaki et al., 2016). A clinical study showed that patients with the best long-term ELN responses have the best long-term results, and patients who received imatinib 800, dasatinib, or nilotinib have a greater chance of attaining optimum response at various periods (Jain et al., 2016).

According to Issa et al. during TKI therapy, if non-Y CCA/Ph¯ (clonal chromosomal abnormalities in Philadelphia chromosome-negative) is detected, physicians should be alerted to the possibility of poor survival outcomes, which may need careful monitoring. For most indices of response and long-term survival, patients with CCA/Ph¯ exhibited a trend toward inferiority. Early response assessment, such as BCR-ABL, was found to have a substantial predictive influence on the multivariate analysis. Patients with CCA/Ph¯ were less likely to get this reaction (74 vs. 81%). For individuals with monosomy 7, the emergence of nondeletion Y CCA/Ph¯ following therapy with different TKI is linked to reduced FFS, EFS, TFS, and OS, as well as a low but substantial chance of developing MDS or AML (Issa et al., 2017; Sasaki et al., 2018).

### CML Patients Receiving TKIs and COVID-19

COVID-19 patients have been compared to an age, sex, and condition matched control group to determine the therapeutic effects of TKIs on their outcome (Başcı et al., 2020). Neither CP CML nor BCR-ABL TKIs induces clinically significant immune suppression, and there is no evidence that CP CML patients are at greater risk of complications by SARS-CoV-2 than the normal community. The clinical diagnosis of COVID-19 is not worse in TKI-treated CML patients than it is in the non-TKI-treated individuals. TKIs should be studied in wide-scale prospective and randomized studies to see if they improve COVID-19 outcomes (Başcı et al., 2020).

The clinical parameters of various TKI drugs used to treat CML are shown in Table 1 (Gleevec, 2008; Sprycel, 2010; Iclusig, 2016; Bosulif, 2017; Tasigna, 2017).

**TABLE 1:** Clinical parameters of different TKIs approved by FDA to treat CML.

Parameters		Imatinib	Dasatinib	Nilotinib	Bosutinib	Ponatinib
Generation		first	second	second	second	third
FDA approval		2001	2006	2007	2012	2012
Molecular formula		C_29_H_31_N_7_O	C22H26ClN7O2S	C28H22F3N7O	C26H29Cl2N5O3	C29H27F3N6O
Dosage	CP	400 mg OD	100 mg OD	300 mg BD	500 mg OD	45 mg OD
	AP/BC	600 mg OD	140 mg OD	400 mg BD		
Plasma bound		95%	96%	98%	94%	99%
Metabolism		They are primarily metabolized by CYP3A4				
T_max_ (hours)		2–4	0.5–6	3	3–6	6
T_1/2_ (hours)		18	3–5	17	22–27	22
Elimination		The major route of drug elimination is through feces and a small amount of urine				
CYP3A4 inducers		When coadministered, drug exposure (AUC and Cmax values) gets decreased				
CYP3A4 inhibitors		When coadministered, drug exposure (AUC and Cmax values) gets increased				
Sensitivity for T315I mutation^a^		>6,400	>200	>2,000	1,890	11
Potency against BCR-ABL		1-fold	325-folds	30-folds	100-folds	400-folds
T315I Resistant		Yes	Yes	Yes	Yes	No

a Sensitivity of BCR-ABL T315I mutation against TKIs (IC_50_ in nM), *in vitro* (Balabanov et al., 2014).

### Adverse Events Associated With TKI Therapy

Imatinib is generally well absorbed, with far fewer complications than traditional drug treatment. The percentage of individuals with CML in CP that had neutropenia and thrombocytopenia of grade 3/4, is approximately 13 and 7%, respectively. In children, 27 percent of patients had neutropenia, 5 percent had thrombocytopenia, and 2.5 percent had anemia, all of grade 3 or 4. By temporarily halting imatinib, the cytopenia is usually fairly controllable. Gastrointestinal disorders like diarrhea, nausea, and vomiting are examples of nonhematologic toxicity. Bone pain, edema, rashes, myalgia and elevated levels of ALT and AST, etc., are the further side effects (Millot et al., 2011).

## Challenges and Resistance to TKI Treatment in CML

Although TKIs are found to be one of the effective targeted chemotherapy for treatment of individuals with CML. But every drug has some toxic responses against the body mechanism or metabolism that has to be overcome either by drug dose modifications or, in some cases, by permanently discontinuing the drug. Similar challenges and actions that have to be taken under treatment of CML using TKI are depicted in Table 2 (Gleevec, 2008; Sprycel, 2010; Iclusig, 2016; Bosulif, 2017; Tasigna, 2017).

**TABLE 2:** The adverse conditions and dose modifications of TKIs during CML treatment.

TKIs	TKI drug response		Action to be taken
Imatinib	CP CML	ANC <1.0 × 10^9^/L and/or Platelets <50 × 10^9^/L	Withhold until ANC ≥1.5 × 10^9^/L and platelets ≥75 × 10^9^/L; then resume at a lower dose
	AP/BC CML	ANC <0.5 × 10^9^/L and/or Platelets <10 × 10^9^/L	Withhold until ANC ≥1 × 10^9^/L and platelets ≥20 × 10^9^/L; then resume at a lower dose
Dasatinib	CP CML	ANC <0.5 × 10^9^/L and/or Platelets <50 × 10^9^/L	Withhold until ANC ≥1.0 × 10^9^/L and platelets ≥50 × 10^9^/L; then resume at a lower dose. Permanently discontinue if AE persists
	AP/BC CML	ANC <0.5 × 10^9^/L and/or Platelets <10 × 10^9^/L	Withhold until ANC ≥1 × 10^9^/L and platelets ≥20 × 10^9^/L; then resume at a lower dose
Nilotinib	ANC <1.0 × 10^9^/Land/or Platelets <50 × 10^9^/L		Withhold until ANC ≥1.0 × 10^9^/L and platelets ≥50 × 10^9^/L; then resume at a lower dose
	ECG with QTC >480 msec		Withhold until QTC <450 msec, then resume with the initial dose
	Increased serum lipase/amylase, bilirubin, hepatic transaminases ≥ Grade 3 and other hepatic impairment		Withhold until AE disappears then resume with a lower dose. Slowly escalates to initial dose if tolerable
Bosutinib	ANC <1.0 × 10^9^/L and/or Platelets <50 × 10^9^/L		Withhold until ANC ≥1.5 × 10^9^/L and platelets ≥75 × 10^9^/L; then resume at a lower dose
Ponatinib	ANC <1.0 × 10^9^/L and/or Platelets <50 × 10^9^/L		Withhold until ANC ≥1.5 × 10^9^/L and platelets ≥75 × 10^9^/L; then resume at a lower dose
	Elevated AST/ALT (Grade ≥2), Elevated serum lipase (Grade 3/4), Grade 3 pancreatitis		Withhold until AE disappears then resume at a lower dose. Permanently discontinue if AE persists
	Elevated AST/ALT (Grade ≥2) concurrent with Elevated Bilirubin and ALP (Grade ≥1), Grade 4 pancreatitis		Permanently discontinue

Even with more effective second-and third-generation TKIs, many CML individuals have developed primary (5–10%) or secondary (20–30%) intolerance and thus become resistant to TKI therapy. Secondary resistance is most commonly caused by acquired Abl-kinase domain (Abl-KD) alterations, which can be noticed in roughly 60% of instances; nevertheless, the most of primary resistance pathways are still unknown (Bower et al., 2016; Russo et al., 2020). The 6-year review of the IRIS study revealed that imatinib therapy is ineffective in around one-third of patients because of 1° or 2° resistance. Approximately 40–90 percent of individuals who are resistant to imatinib have BCR-ABL mutation, based on the cancer stage and detection equipment’s sensitivity. T315I is an especially important mutation as it is quite prevalent (15–20%) and causes tolerance to almost all TKIs currently used in clinical practice, except ponatinib. It is found in ABL kinase domain’s ATP-binding region and provides one of imatinib’s six binding sites. This binding site is destroyed when threonine is replaced by isoleucine, as well as a structural impediment that prevents imatinib, nilotinib, dasatinib, and bosutinib from reaching it (Hughes et al., 2014). Imatinib resistance can be caused by either primary or secondary causes. Pharmacokinetic changes, such as anomalies in transport or drug efflux, may cause primary resistance. Because of BCR-ABL mutations or the involvement of salvage mechanisms, patients are more prone to develop secondary or acquired resistance. The T315I mutation is the most dreaded mutation since it is resistant to all the TKIs that have been approved by FDA for this disease. Several drugs, such as ponatinib, are being tested in clinical studies to block kinase activity linked to T315I (O’Hare et al., 2009). According to prior studies, TKI can restore the levels of specific miRNAs, and this mechanism can mediate the effects of TKIs on CML cells (Bugler et al., 2019).

CML is divided into three stages, referred to as CP, AP, and BP. With the introduction of TKIs and the subsequent success of TKI cessation, a fourth-phase, treatment-free remission (TFR-CML), has developed. In the absence of CML-directed treatment, this phase begins after TKI therapy is stopped and is defined by sustained remission with undetectable (or very low detectable) illness by the most sensitive technique of testing. This phase differs from CP since there is no additional molecular, cytogenetic, or hematological development. A clinical study revealed that patients were treated with TKIs for 11 years, and MMR and MR4.5 were maintained for 10 and 5.5 years, respectively, until therapy was discontinued. As a result, the rapid transition to BP CML was not preceded by CP development, and this sudden transformation had been reported in fewer than 1% of the CML individuals undergoing imatinib therapy. Cellular immunity, in the form of natural killer cells and cytotoxic T lymphocytes, may have a role in TFR-CML, according to one theory. This rapid blastic change might be the outcome of a lack of immune monitoring or a disease breakout (Alfayez et al., 2019). But it might also be related to some genetic and epigenetic abnormalities that require further clinical research for validation.

Clinical study data show that epigenetic medication treatment can produce objective responses in CML patients, even in later stages and amid imatinib resistance. Though, these reactions are rarely long-lasting, necessitating the pursuit of alternative recovery methods. Not only are many TKI options available (e.g., Imatinib, nilotinib, dasatinib, ponatinib, and bosutinib), but novel agents (e.g., ABL001) are being investigated, and for individuals with chronic or non-responsive illness, allogeneic transplantation remains a surgical option.

Given below are the possible reasons/mechanisms for TKI resistance (Balabanov et al., 2014).

1. BCR-ABL Dependent
  i. Overexpression of BCR-ABL kinases as a result of amplification leads to a TKI-resistant scenario.
  ii. Mutations in BCR-ABL resist TKI binding as a result of conformational change in ABL.
2. BCR-ABL Independent
  i. Initiation of other compensatory intracellular signaling pathways (like LYN, HCK), due to which, despite successful suppression of the primary oncogenic driver kinase, cells continue to proliferate and thus results in TKI resistance.
  ii. The overexpression of efflux transporters, like ABCB1 (MRD-1, for which imatinib acts as a substrate), results in a decrease of TKI levels in cells.
  iii. The downregulation of influx transporters, like OCT1 (in the case of imatinib), also results in reduced efficacy of TKI and finally leads to TKI resistance.

### An Epigenetic Mechanism of Imatinib Resistance Associated With HOXA4 Gene Promotor

The inhibition of the HOXA4 protein may interfere with the normal growth and proliferation of myeloid progenitors. Imatinib resistance in CML patients may be caused by an epigenetic mechanism in the BCR-ABL-independent pathway. The hypermethylation of HOXA4 gene appears to be inhibiting the clinical response to IM, and as a result, is acting as a significant inhibitor of normal leukemogenesis. Inhibition of this process might be a superior treatment option, necessitating hypomethylating drugs in CML patients with this epigenetic resistance mechanism. As a result, apart from BCR-ABL gene mutation analyses, the hypermethylation profile of the HOXA4 gene might be used as an epigenetic biomarker in CML patients for predicting reaction to imatinib treatment (Elias et al., 2013).

According to recent research, the long noncoding RNA (lncRNA) HOTAIR (HOX transcript antisense RNA) is involved in the development of both solid tumors like breast cancer and NSCLC, as well as hematopoietic malignancies like AML. The epigenetic regulatory mechanisms of HOTAIR in advanced CML, on the other hand, remain unknown. Li et al. suggested that demethylation drugs may provide a novel remedy for CML-BC, as HOTAIR is a possible therapeutic target (Li and Luo, 2018).

### Second Malignancies Incidence in CML Patients

In the age of TKIs, the incidence of second malignancies in CML patients has increased. Sasaki et al. (2019), reported data of newly diagnosed CML patients in 2001–2014, and it was found that 5.6% of the cancer patients had more than one malignancy after the CML diagnosis. The most prevalent sites of second cancers were the male genital system, digestive system and respiratory system. CML-CP had a slightly higher relative incidence of total second malignancies than the general population, with a modest rise in the AER (Sasaki et al., 2019).

### The Epigenetic Mutation Associated With Cardiovascular Events in CML Patients

There have been reports of cardiovascular or arteriothrombotic adverse events (CV- or AT-AEs) in CML patients receiving TKI therapy. The second or third-generation TKIs, such as ponatinib, significantly increase the risk of CV-AEs (excluding hypertension) and AT-AEs in CML patients (Jain et al., 2019). Myelopoeisis epigenetic regulation is controlled by the *TET2* gene, which codes for the protein TET2 methylcytosine dioxygenase. Through the oxidation of methyl-cytosine, TET proteins play a role in DNA alteration, also in normal and malignant growth. A DNA epigenetic modification termed as hydroxymethylation is catalyzed by Tet2. As a result, it had been postulated that Tet2 regulated gene expression in macrophages exposed to excessive cholesterol. As a result, somatic mutations in hematopoietic cells have a role in the onset of atherosclerosis in humans. It was suggested that clonal hematopoiesis might be a controllable risk factor, either by using cholesterol-lowering medicines or targeting certain inflammatory processes (Jaiswal et al., 2017).

### Role of Cytogenetic Abnormalities in Prognosis

Patients with severe CML are more likely to have additional cytogenetic abnormalities (ACAs) in addition to the Philadelphia chromosome. Long-term survival may be improved by combining TKI treatment with systemic chemotherapy. No matter what the blast count is, the existence of CBF translocations determines whether or not a patient has AML, because CBF rearrangements are seldom diagnosed as ACAs. In individuals with CML, CBF rearrangements as ACAs might be regarded as high-risk characteristics (Morita et al., 2021a).

## Epigenetic Emerging Therapies for CML in Combination With TKIs: Efficacy and Challenges

There is significant evidence that the accumulation of various genetic and epigenetic abnormalities, in addition to the BCR/ABL1 oncoprotein, influences disease progression, treatment response, and overall clinical prognosis in CML patients. The development of a variety of illnesses has been related to DNA hydroxymethylation. Disease development, drug reaction, and clinical outcome of different diseases are all influenced by the DNA hydroxymethylation of gene promoters (Jelinek et al., 2011). The previous results of the clinical studies suggest that in CML patients undergoing imatinib treatment, hydroxymethylation of gene promoters of cell cycle regulators and autophagy genes may be a significant predictor of tumor growth, identifies inefficient imatinib responders, and indicates a relatively poor treatment outcome. As a result, the findings encouraged the use of demethylating agents in conjunction with TKI treatment to improve clinical consequences (Jelinek et al., 2011). The previous studies linking hypermethylation of SOCS1 gene to downregulation of expression suggested that the lack of negative cytokine signaling caused by SOCS1 protein epigenetic suppression might contribute to CML development. Small molecules with two src homology domains make up SOCS proteins. As a result, SOCS1 expression may be a new and valuable marker for CML therapeutic follow-up, as well as a new therapeutic strategy for developing successful CML target therapies (Liu et al., 2003).

Over the last 20 years, the invention of TKIs to counteract the BCR-ABL protein’s constitutive tyrosine kinase function has greatly increased disease control and clinical outcomes. However, most patients are not healed, and designing treatment strategies that target epigenetic pathways is a potential way of increasing cure rates. During the formation and growth of CML, various epigenetic pathways are changed or reprogrammed, leading to changes in histone modifications, DNA methylation, and transcriptional dysregulation (Bugler et al., 2019). Consequently, with the advent of TKIs as the primary mode of therapy, the occurrence of CML has risen, rendering it a manageable, chronic condition. Although TKI therapy has revolutionized CML treatment, 25–30 percent of patients with CP-CML have failed TKI treatment, half had mutations in the BCR–ABL1 kinase domain and 50 percent of patients are unknown for the failure of this therapy (Rohrbacher and Hasford, 2009; Baccarani et al., 2013). Potentially harmful somatic mutations of epigenetic modifiers are common in CP-CML at the time of diagnosis, and when combined with other factors associated, they can be useful predictive indicators to identify the best TKI for every patient (Nteliopoulos et al., 2019). The chemotherapy resistance during treatment might occur at any stage, and thus the emergence of drug resistance remains a major issue.

### Combination Therapy Using TKI With Other Drugs/Inhibitors: An Epigenetic Reprogramming

**FIGURE 1:**
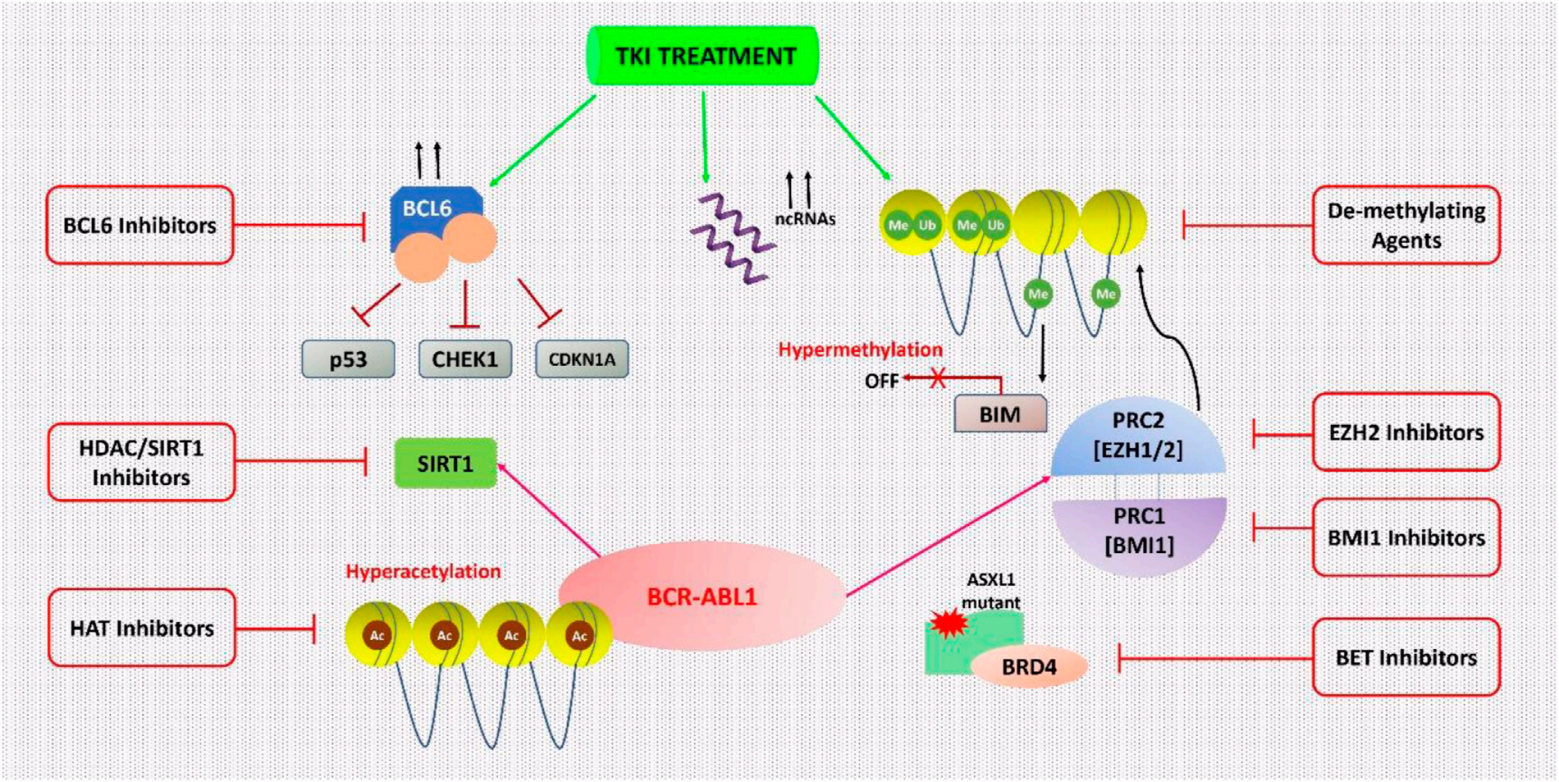
Epigenetic processes in CML cells are being targeted. CML cells are epigenetically reprogrammed via several mechanisms. This dysregulation can occur only in the presence of TKI therapy in various cases. With this understanding, many epigenetic treatments were suggested to inhibit these mechanisms and to result in the complete destruction of CML cells especially in combination with TKI therapy.

In 1983, after the discovery of the first cancer-epigenetic reprogramming relations, emerging evidence indicates that genetic and epigenetic changes cause cancers and that some of them occurring before profound leukemia commences preleukemia (Feinberg et al., 2006; Feinberg and Vogelstein, 1983). The BCR-ABL1 mutation induces epigenetic reprogramming, which is important in CML, as well as converting HSCs to LSCs. Polycomb-group (PcG) proteins are one kind of epigenetic regulator that is believed to be disrupted in CML LSC (Di Carlo et al., 2019). There are two complexes in PcG proteins, namely PRC1 and PRC2 (Polycomb Repressive Complex). Given the number of studies that PRC2 plays a role in cancer (including solid tumors and multiple myeloma), it’s no surprise that a variety of therapeutics targeting this complex have appeared and are under clinical studies of Phase I and II (Fioravanti et al., 2018; Gulati et al., 2018). In comparison to TKI therapy alone, Scott et al. demonstrated significant targeting of CML stem cells using the EZH2 (a core component of PRC2) inhibitor Tazemetostat in conjunction with TKI. As a result, combined therapy may be a new clinical option for CML treatment, as shown in Figure 1 (Scott et al., 2016).

Sirtuin 1 (SIRT1) is a NAD-dependent HDAC that acts on the nonhistone target p53 in CD34^+^ CML cells and has been associated with leukemogenesis and the persistence of CML LSCs. After imatinib therapy, SIRT1 expression significantly decreased but not completely depleted in CML cell lines, paving the way for effective SIRT1 inhibition. The treatment with the SIRT1 inhibitor (e.g., Selisistat or EX-527) via activation of p53 signaling, improved the effect of TKI treatment (Chen and Bhatia, 2013; Li et al., 2012; Yuan et al., 2012). SIRT1’s function is one of the most prevalent causes of treatment resistance in several malignancies, including CML (S et al., 2021).

BCL6 is a transcription factor that can epigenetically control a variety of its targets by altering the accessibility of chromatin at promoter and enhancer sites. It is often mutated in lymphoma cells (Hatzi et al., 2013). BCL6 expression is modest in TKI-naive CML cells, but it is substantially increased in CML cell lines and primary CD34+ cells after TKI therapy, although its function in CML is unknown (Hurtz et al., 2011). In Ph+ ALL patients, the BCL6 peptide inhibitor, RI-BPI was coupled with imatinib to avoid TKI resistance and to enhance imatinib’s efficacy (Duy et al., 2011). In CD34^+^38- CML LSCs, the use of RI-BPI increased the effectiveness of imatinib treatment, and decreased the ability to form CML cell colony, thus eliminated CD34^+^38- LSCs by enhancing programmed cell death. Other BCL6 small molecule inhibitors, such as FX1, significantly reduced CML CD34^+^ cells’ colony-forming ability, in combination with TKI (Madapura et al., 2017).

It had been suggested by San José-Eneriz et al. (2009), that the concomitant use of imatinib and a de-methylating drug such as 5-aza-2′-deoxycytidine (Decitabine) might result in better outcomes in individuals with reduced expression of the pro-apoptotic BCL-2-interacting mediator (BIM). This downregulation is epigenetically mediated by BIM methylation in most CML individuals and has a negative impact on health (San José-Eneriz et al., 2009). TKI-resistant leukemic stem cells (LSCs) are still a big problem in CML, and finding ways to get rid of them is a major unsatisfied clinical need. Scott et al. (2016) showed that for the existence of LSC, EZH2 and H3K27me3 reprogramming is essential, nevertheless, it also makes LSCs vulnerable to the mutual effects of EZH2 inhibitor and TKI. That becomes a novel strategy for more efficiently targeting LSCs in TKI receiving patients (Scott et al., 2016).

### Concomitant Use of TKI and DNMT Inhibitor (a Class of Epigenetic Drugs)

**FIGURE 2:**
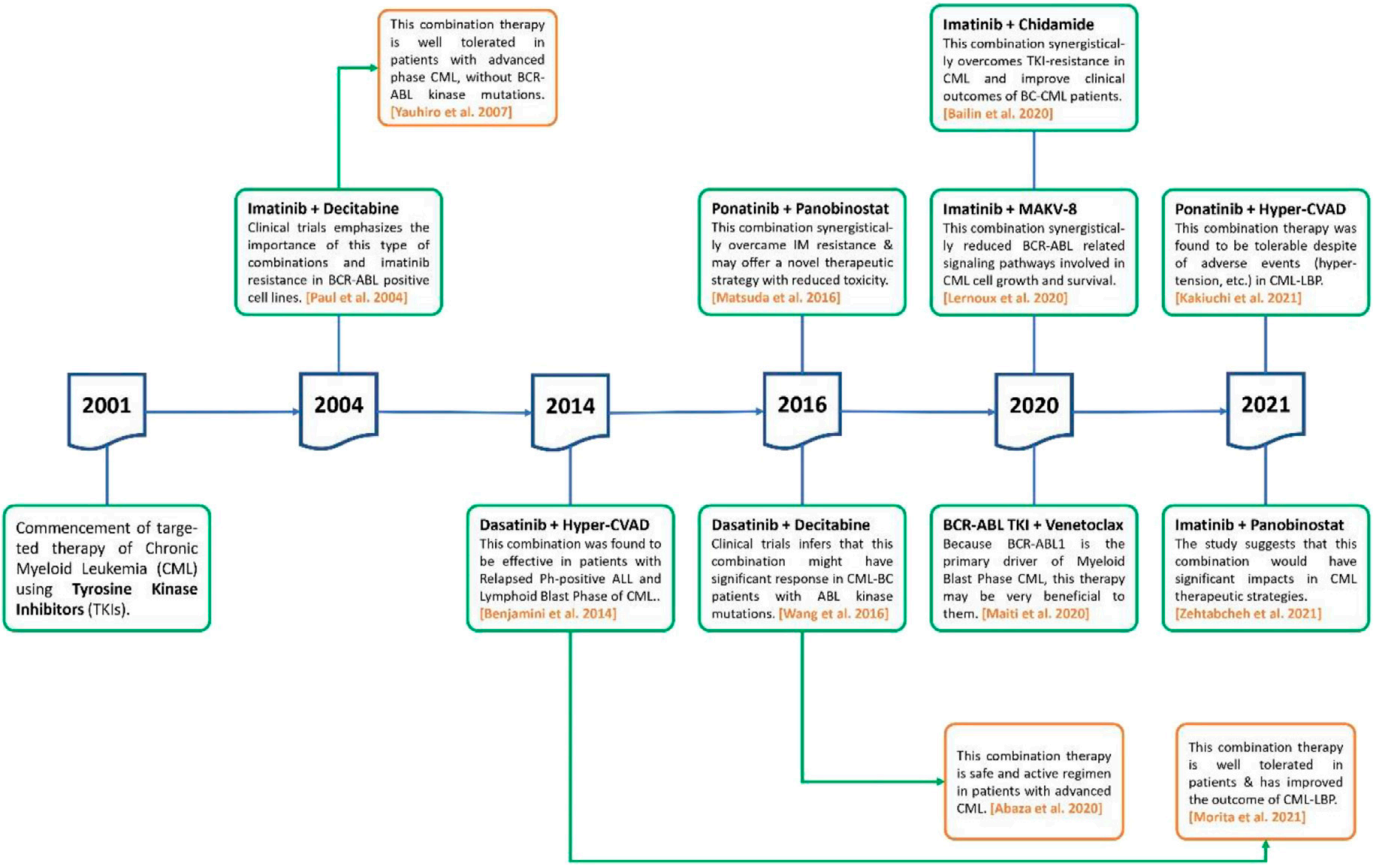
The role of TKIs in combination with epigenetic drugs to treat CML during last 2 decades.

DNMT inhibitors are cytosine analogs that bind to DNA and inactivate genes, inhibiting the methyltransferase enzyme reversibly. Azacytidine and decitabine are DNMT inhibitors that had been approved by the FDA for the treatment of myelodysplastic syndrome (MDS). Low doses of these inhibitors are effective, but high doses are lethal (Yang et al., 2006). Decitabine is a cytosine derivative that is used as an intravenous antineoplastic agent in the treatment of MDS. It receives FDA approval in 2006 for the treatment of MDS and was also approved by the EU for the treatment of AML. The strategy of concomitant use of TKIs with epigenetic drugs for CML therapy was initiated after the approval of the first tinib drug (imatinib) in 2001 (Figure 2).

#### Imatinib Plus Decitabine

In CML patients with AP or BC phase, a Phase II trial of low-dose decitabine in conjunction with an active metabolite of imatinib (i.e., imatinib mesylate, IM) was performed. Imatinib 600 mg OD and decitabine 15 mg/m^2^ intravenously (5 days a week) were given to patients for 2 weeks. The major and modest cytogenetic responses were reported which includes: 32% patients with complete and 4% patients had moderate hematologic responses, while 7% patients had hematological development. The individuals who lack BCR-ABL kinase mutations had a greater hematologic response rate (53%) than those with mutations (14%). The median hematologic response lasted 18 weeks. The most common side effect was myelosuppression, which resulted in neutropenic fever in 32% of individuals. So, in AP CML without BCR-ABL kinase mutations, it was concluded that decitabine with imatinib in combination treatment, is well tolerated and effective (Oki et al., 2007).

In recent years, the reprogramming of mitochondrial metabolism has been identified as a hallmark of cancer, including CML, and could be used for medicinal purposes. The impacts of various drugs on mitochondrial activity in the CML cell line K562 were explored, and it was shown that decitabine in combination with 5-aminoimidazole-4-carboxamide ribotide (AICAR) might efficiently boost ATP content and mitochondrial biogenesis. Furthermore, it has been shown that AICAR-decitabine therapy increased the sensitivity of the K562 cells for imatinib. Based on these findings, it is clear that TKIs coupled with mitochondrial modulation may give treatment strategies for CML treatment (Zhu et al., 2020).

#### Dasatinib Plus Decitabine

Treatment for AP CML still lacks, as single-agent TKIs exhibit weak and short-lived action. A phase I/II study was undertaken in patients with advanced CML to see if the concomitant use of dasatinib and decitabine was safe and effective. Two distinct dosing regimens were tested with a beginning decitabine dose of either 10 or 20 mg/m^2^ OD for 10 days with dasatinib 100 mg OD. Decitabine 10 or 20 mg/m^2^ OD for 10 days, with dasatinib 140 mg OD, was the target dosage. There was no dose-limiting toxicity identified with either regimen at the initial dosages. In 93% of individuals, treatment-emergent hematological adverse effects of grade 3 were observed. Meanwhile, 48% of patients had a major hematopoietic response, whereas 22% had a weak hematologic reaction, with 44% of them having a large cytogenetic reaction and 33% having a significant biomolecular response. Decitabine with dasatinib is a safe, innovative, and effective treatment for advanced CML, with a life expectancy among patients that looks to be higher than with either agent alone (Abaza et al., 2020).

#### Venetoclax Plus BCR-ABL TKIs

As a BCL2 inhibitor, venetoclax is authorized for use in aged or “unsuitable” individuals with newly diagnosed AML, together with low-dose cytarabine or HMA. Venetoclax has demonstrated preclinical efficacy against TKI-resistant CML cells and synergy with BCR-ABL TKI in eliminating leukemic stem cells in advanced CML In CML-MBP, dasatinib is effective as a single treatment; however, the venetoclax-TKI combination may be more effective than dasatinib alone. Ponatinib has shown promising results in advanced Ph+ leukemias and relapsed/refractory pre-B ALL whether used alone or in conjunction with nonchemotherapeutic treatments. Ponatinib synergizes with venetoclax in Ph+ ALL samples by inducing LYN-mediated pro-apoptotic BIM, reducing anti-apoptotic MCL1, and perhaps removing venetoclax resistance. The combination of venetoclax and ponatinib in this patient population is therefore supported by clinical trials. Thus, treatment of advanced Ph+ myeloid leukemias with venetoclax and TKI-based combination regimens is a viable option. Because BCR-ABL1 is the primary factor of CML-MBP, this combination could be very beneficial to them (Maiti et al., 2020).

Crawford et al. (2009) observed that a rise in proteasome proteolytic activity is linked to BCR/ABL genetic and epigenetic alterations, and inhibiting the proteasome might cause extremely higher cell apoptosis in BCR/ABL positive cells than in BCR/ABL negative cells. The combined inhibition of HDACs and the proteasome leads to a significant decrease in both efficacy and metabolism of CML-derived BCR/ABL-expressing K562 cells than either drug alone, indicating that treatment with concomitant use of HDAC and proteasome inhibitors could be a successful approach in CML therapy. According to Jagannath et al. (2010) in relapsed/refractory multiple myeloma patients, the clinical therapy with the combined use of HDAC and proteasome inhibitors showed promising anticancer activity (Jagannath et al., 2010).

### Concomitant Use of TKI and IC/HMA in Myeloid BP-CML Patients

It has been demonstrated that TKI + IC/HMA (intensive chemotherapy/hypomethylating agent) therapy provides greater response rates, reduced recurrence risk, and superior 5-years EFS/OS than TKI alone, suggesting that rather than TKI alone, the combination therapy with IC or HMA + TKI (especially received decitabine) should be taken into account for the appropriate frontline therapy for such patients. Long-term survival rates were similar between IC + TKI and HMA + TKI, with a long-term survival rate of around 30% and improved outcomes for patients who were able to undergo ASCT (allogeneic stem cell transplant). However, while being less intensive than an IC method, the combination of HMA and second/third generation TKI is extremely successful (Saxena et al., 2021). As a result of their poor prognosis, patients with CML-BP require novel treatment options (Jain et al., 2017). It was found that the use of hyper-CVAD (cyclophosphamide, vincristine sulfate, doxorubicin hydrochloride/adriamycin, and dexamethasone) with dasatinib therapy, the outcome of CML-lymphoid BP has improved, and the survival rate is equivalent to that of Ph+ ALL. Using new TKIs and targeted agents, further improvements may be made in the future (Morita et al., 2021b).

### Concomitant Use of TKI and HDAC Inhibitor (a Class of Epigenetic Drugs)

HDAC inhibitors are chemical compounds that inhibit histone deacetylases. They have a long tradition of use as mood stabilizers and anti-epileptics in psychology and neurology. They’ve recently been studied as potential cancer, viral, and infectious disease therapies (Miller et al., 2003; Blanchard and Chipoy, 2005; Mwakwari et al., 2010; Patil et al., 2010). HDAC inhibitors are derived from natural and synthetic compounds of varying target specificity and actions. HDAC inhibitors are divided into four categories: hydroxamic acids (or hydroxamates), cyclic tetrapeptides, benzamides and short-chain fatty acids. Due to the concept that BCR/ABL signaling and epigenetic changes might act as two major reasons for disease and tolerance, the combined effects of BCR/ABL inhibition utilizing imatinib with panobinostat (a multi-HDAC inhibitor) in K562 cells were studied (Housman et al., 2014). In CML-derived K562 cells, the combination of panobinostat with imatinib showed synergistic antineoplastic responses and improved therapeutic effectiveness, as predicted (Zehtabcheh et al., 2021).

#### Imatinib Plus MAKV-8

MAKV-8 has been first reported for its IC_50_ of 2 pM, *in vitro*, against HDAC3 and HDAC6 and its anti-proliferative effect for pancreatic tumor cells, which was analogous to SAHA. It comprises the 6-methylene linker and a CAP group containing arylisoxazole. The chemical MAKV-8 is a potent pan-HDAC inhibitor *in vitro* and also in several cell lines of CML. In addition, in imatinib-sensitive/resistant BCR-ABL-positive CML cells, MAKV-8 in combination with imatinib displayed substantial anti-tumor properties, while healthy cell types exposed to this co-therapy showed relatively minimal impact. BCR-ABL expression and phosphorylation, as well as downstream targets expression important in CML growth and survival, were all inhibited by MAKV-8-imatinib combination (Lernoux et al., 2020).

#### Imatinib Plus Panobinostat

Investigation of the combined effects of BCR/ABL inhibition with Imatinib and the multi-HDAC inhibitor panobinostat was performed in K562 cells The combination of panobinostat with imatinib was reported to have synergistic antileukemic effects and increased treatment effectiveness in K562 cells generated from CML patients (Zehtabcheh et al., 2021).

#### Ponatinib Plus Panobinostat

The concomitant use of panobinostat with ponatinib to treat IM resistance which was caused by either gene amplification or T315I mutation in BCR-ABL was found to be effective. This synergetic impact could just be because of the kinase activity suppression of ABL accompanied by BCR-ABL protein degradation. The concomitant use of these drugs could lead to a novel treatment approach with outstanding anti-CML efficacy and few side effects. The XTT test was used to assess the growth inhibitory effects of IM, panobinostat, and ponatinib alone or in combination on K562 cells, K562/IM-R1 cells, Ba/F3 cells, and Ba/F3/T315I cells. K562/IM-R1 cells 12 times, while Ba/F3/T315I cells were shown to be 13 times more IM-resistant than parental counterparts in this experiment. Importantly, as compared to either drug alone, the panobinostat and ponatinib combination demonstrated a significantly greater inhibitory growth impact on all cell lines (Table 3) (Matsuda et al., 2016).

**TABLE 3:** This data has been extracted from Matsuda et al. (2016). For 72 h, cells were exposed to the same concentration of ponatinib, panobinostat, or their combination, before being tested using XTT assay.

Drug/s	CML cell lines (IC_50_ values in µM)			
	K562	K562/IM-R1	Ba/F3	Ba/F3/T315I
Ponatinib	2	3	5	30
Panobinostat	50	51	40	47
Combination	0.7	1.3	3.7	10

#### Imatinib Plus Chidamide

Chidamide is a new selective HDAC inhibitor that has demonstrated its efficacy to treat hematological cancers. Chidamide reduced the development of the CML cell, causing apoptosis and arrest of the cell cycle when used alone. Furthermore, in the CML cell line KBM5, as well as IM-resistant CML cells KBM5 bearing T315I mutations, chidamide in conjunction with imatinib induced synergistic fatality, with a significant decrease in kinase activity of BCR-ABL and expression of acetyl-histone H3. In IM-resistant cells, the combined therapy significantly reduced the fundamental activity of β-catenin signaling and eliminated the mesenchymal stromal cells (MSCs) shielding effects on CML cells. As a result, this combination therapy is likely to be a feasible way to treat TKI-resistant CML and improve treatment strategies with BC-CML (Table 4) (He et al., 2020).

**TABLE 4:** The combination therapy for CML using TKIs with epigenetic drugs.

S.No.	TKIs	Epigenetic drug	Response
1	Imatinib	Decitabine	Well tolerated and effective
2	Dasatinib	Decitabine	Safe, innovative and effective
3	Imatinib/Dasatinib/Nilotinib/Ponatinib	Venetoclax	Beneficient and effective
4	Imatinib/Dasatinib/Nilotinib/Ponatinib	IC/HMA, Hyper-CVAD	Extremely successful and improved survival rate
5	Imatinib	MAKV-8	BCR-ABL expression and phosphorylation inhibited
6	Imatinib	Panobinostat	Synergistic antileukemic effects
7	Ponatinib	Panobinostat	Significantly greater inhibitory growth impact on all cell lines
8	Imatinib	Chidamide	Induce synergetic fatality

## Conclusion and Future Prospects

Today, one of the finest examples of effective targeted therapy in CML is that the survival rate of CML patients treated with TKIs seems to be relatively close to the healthy individuals. Since the development of first and second-line TKIs, substantial progress in CML therapy has been accomplished. Despite the TKI treatment’s excellent efficacy, some patients developed resistance owing to BCR-ABL kinase domain alterations, resulting in therapeutic failure. The development of CML and its resistance to treatments are both influenced by abnormal epigenetic control of key genes. Because epigenetic alterations may be controlled, novel medications that target distinct epigenetic pathways, such as demethylating agents and HDAC inhibitors, have been evaluated for CML treatment, particularly in individuals who have developed imatinib resistance. Finally, recent advances in biotechnology and bioinformatics have created new potential techniques for *de novo* epigenetic factor characterization and greater knowledge of epigenetic pathways, which can further develop tailored CML therapies in the future.

## Glossary

ACAs: Additional cytogenetic abnormalities
AE: Adverse effects
AER: Absolute excess risk
ALL: Acute lymphoblastic leukemia
ALT: Alanine transaminase
AML: Acute myeloid leukemia
ANC: Absolute neutrophil count
AP: Accelerated phase
ASCT: Allogeneic stem cell transplant
AST: Aspartate aminotransferase
AUC: Area under the curve that represents the total drug exposure across time
BC: Blast crisis
BCR: Breakpoint cluster region
BD: Bis in die (two times daily)
BP: Blast Phase
CBF: Core binding factor
CCyR: Complete cytogenetic response
CHR: Complete hematologic remission
Cmax: Maximum serum concentration that a drug achieves after administration
CML: Chronic myeloid leukemia
COVID-19: Coronavirus Disease 2019
CP: Chronic phase
DNMT: DNA methylation
EFS: Event-free survival
ELN: European LeukemiaNet
EMR: Major molecular remission
FDA: Food and drug administration
FFS: Failure-free survival
HDAC: Histone deacetylase
HMA: Hypomethylating agent
HOTAIR: HOX transcript antisense RNA
IC: Intensive chemotherapy
IC_50_: Half maximal inhibitory concentration
LSC: Leukemic stem cell
MDS: Myelodysplastic Syndrome
OD: Omne in die (once a day)
OS: Overall survival
PcG: Polycomb-group
PCyR: Partial cytogenetic response
PRC: Polycomb repressive complex
QTc: QT corrected for heart rate
SAHA: Suberoylanilide hydroxamic acid
SARS CoV-2: Severe acute respiratory syndrome coronavirus 2
SIRT: Sirtuin
SOCS: Suppressor of cytokine signaling
**T** **_1/2_**: Elimination half-life of a drug
TFS: Transformation-free survival
TKI: Tyrosine kinase inhibitor
**T** **_max_**: Time at which the Cmax is observed
WHO: World Health Organization
